# The Association of the Neutrophil-Lymphocyte Ratio With the Outcome of Diabetic Foot Ulcer

**DOI:** 10.7759/cureus.33891

**Published:** 2023-01-17

**Authors:** Manduri Sathvik, Keerthana Vuppuluri, Phanindra Dulipala

**Affiliations:** 1 Department of General Surgery, Katuri Medical Collage and Hospital, Guntur, IND; 2 Department of Community Medicine, Katuri Medical College and Hospital, Guntur, IND

**Keywords:** prognosis, neutrophil-to-lymphocyte ratio (nlr), non-healing ulcers, healing ulcers, diabetic foot ulcers

## Abstract

Introduction

One of the most common lower-extremity impediments in people suffering from diabetes mellitus (DM) is foot ulceration. Neutrophil-lymphocyte ratio (NLR) is a useful measure in predicting disease-specific morbidity and mortality.

Objectives

The objective is to study the association between diabetic foot ulcer healing and the NLR.

Methodology

A prospective analytical study was conducted among 100 patients with diabetic foot ulcers admitted to a surgical ward in a teaching hospital between April and November 2022. Basic demographic details, ulcer examination, and NLR were evaluated on the day of admission, and the status of ulcers was assessed after six weeks and the outcome was compared with the NLR value. Data analysis was done using SPSS version 20 software (SPSS, Inc., Chicago, IL).

Results

The average neutrophils, lymphocytes, and neutrophil-lymphocyte ratio were, respectively, 94.73%, 14.97%, and 6.65%. 58% had healing ulcers, and 42% had non-healing ulcers. 44% of study subjects had NLR <6, which is normal, and 56% had NLR >6, which is abnormal. Among 58 subjects with healing ulcers, 75.9% had NLR < 6, and among 42 subjects with non-healing ulcers, 100% had NLR >6, which was statistically significant. The mean NLR in the healing group was 5.15 and in the non-healing group was 8.205; this was statistically significant. This shows an increased NLR has a predisposition towards non-healing chronic ulcers with a poor prognosis.

Conclusion

NLR can be used as a reliable indicator for determining the healing status of diabetic foot ulcers.

## Introduction

A chronic metabolic condition known as diabetes mellitus (DM) is characterized by constant and continual hyperglycemia. This may be due to reduced insulin secretion, resistance to insulin's peripheral effects, or a combination of the two. An ulcer is the most common complication of diabetes, which is due to microvascular damage. This can lead to both morbidity as well as mortality [1]. Diabetic foot ulcers are one of the most prevalent consequences of poorly treated diabetes mellitus. Diabetic foot ulcers are a prevalent cause of osteomyelitis of the foot and amputation of lower limbs [2-4].

The chances of developing diabetic foot ulcers can be reduced by following simple strategies like regular foot examination, educating the patient regarding foot care, and following simple hygiene practices. The interdisciplinary approach to diabetic foot disorders has been shown to be the best way to obtain favorable limb salvage rates in high-risk diabetic patients [5].

The neutrophil/lymphocyte ratio (NLR) is a low-cost, widely accessible biomarker that has been shown to be effective as both systemic neutrophilia and lymphopenia are associated with a poorer prognosis in a variety of inflammatory, viral, and cardiovascular disorders, as well as malignancies [6-9]. The differential blood cell count test yields the NLR by dividing the neutrophil-to-lymphocyte ratio. When compared to many other wound-specific indicators, including matrix metalloproteinases or growth factors, it is an economical and more practical approach to assessing immune system activity. In addition, NLR is stable and resistant to physiological and environmental changes that impact the outcomes of other markers, such as dehydration, exercise, and blood sample processing [3]. Numerous studies have established its significance in systemic inflammation in diabetes [10,11]. Considering the benefits listed above, this study aimed to determine the association of NLR with the outcome of diabetic foot ulcers.

## Materials and methods

A prospective analytical study was conducted between April and November 2022 in a tertiary care hospital. The study population included all patients admitted to the surgical ward with foot ulcers during the study period. A convenience sampling technique was used. The study sample size was calculated using the formula:

n = Z21 − α/2 × Sp × (1 − Sp) / (1 − p) × d^2^

where Z1 − α/2 is a two-tailed probability for a 95% confidence interval = 1.96; Sp (%) is the specificity of NLR < 4.2 = 71% with a precision of 10% and 95% confidence interval from a study conducted by Vatankhah et al. [3]; d (%) is the precision or allowable error for the specificity of NLR < 4.2 = 0.1; and p (%) is the prevalence of complete wound healing = 0.2.

Substituting the above values in the formula:

N = 1.962 × 0.71 × (1 − 0.71) / (1 − 0.2) × 102

N = 98.87 rounded off to 100

Thus, the total sample size was 100.

Inclusion and exclusion criteria

All known diabetic patients with foot ulcers above 30 years of age with grade 1 (superficial, full-thickness ulcer limited to the dermis, not extending to the subcutis) and grade 2 (ulcers of the skin extending through the subcutis with exposed tendon or bone and without osteomyelitis or abscess formation) ulcers by Meggitt Wagner classification [12], and patients who were willing to take part in the study, were included in the study. Patients with grade 3 (deep ulcers with osteomyelitis or abscess formation), grade 4 (localized gangrene of toes to forefoot), and grade 5 (foot with extensive gangrene) ulcers by the Meggitt Wagner classification, patients with systemic diseases like ischemic heart disease, patients with venous ulcers, and critically ill patients were excluded from the study.

All of the patients who satisfied the inclusion and exclusion criteria were included in the study until the desired sample size of 100 was attained. On the day of admission, neutrophil and lymphocyte counts were obtained using the manual counting method, from which the neutrophil-lymphocyte ratio was calculated. A clinical examination of the ulcer was done, and a daily hydrogel dressing was done till the patient was discharged from the hospital. The average admission time was one week. After six weeks, the ulcer was examined clinically for the development of red granulation tissue at the base of the ulcer, and the healing status of the ulcer was determined. The relationship between outcome and NLR was determined. Ethical clearance was obtained from the Institutional Ethics Committee (IEC), and informed consent was obtained from each study subject. Data collected were entered into an Excel sheet, and the results were expressed in percentages. Data analysis was done using SPSS version 20 software for the chi-square test and independent t-test.

## Results

The present study included 100 patients with diabetic foot ulcers, ranging in age from 40 to 75 years, and the mean age of the study population was 57.1 ± 11.289 years. The mean duration of diabetes in the study subjects was 9.49 ± 3.03 years, ranging between 5 and 15 years. Age-wise distribution, gender distribution, duration of diabetes, HbA1C levels, and any history of trauma prior to the onset of the ulcer were shown in Table 1.

**Table 1:** Demographic details, duration of diabetes, HbA1C levels, history of trauma.

Variable	Percentages (%)
Age in years	
40-45	23
46-50	13
51-55	7
56-60	12
61-65	16
66-70	15
71-75	14
Gender	
Male	42
Female	58
Duration of diabetes	
<10 years	54
≥10 years	46
HbA1C levels	
<7	53
>7	47
History of trauma	
Yes	53
No	47

**Figure 1:**
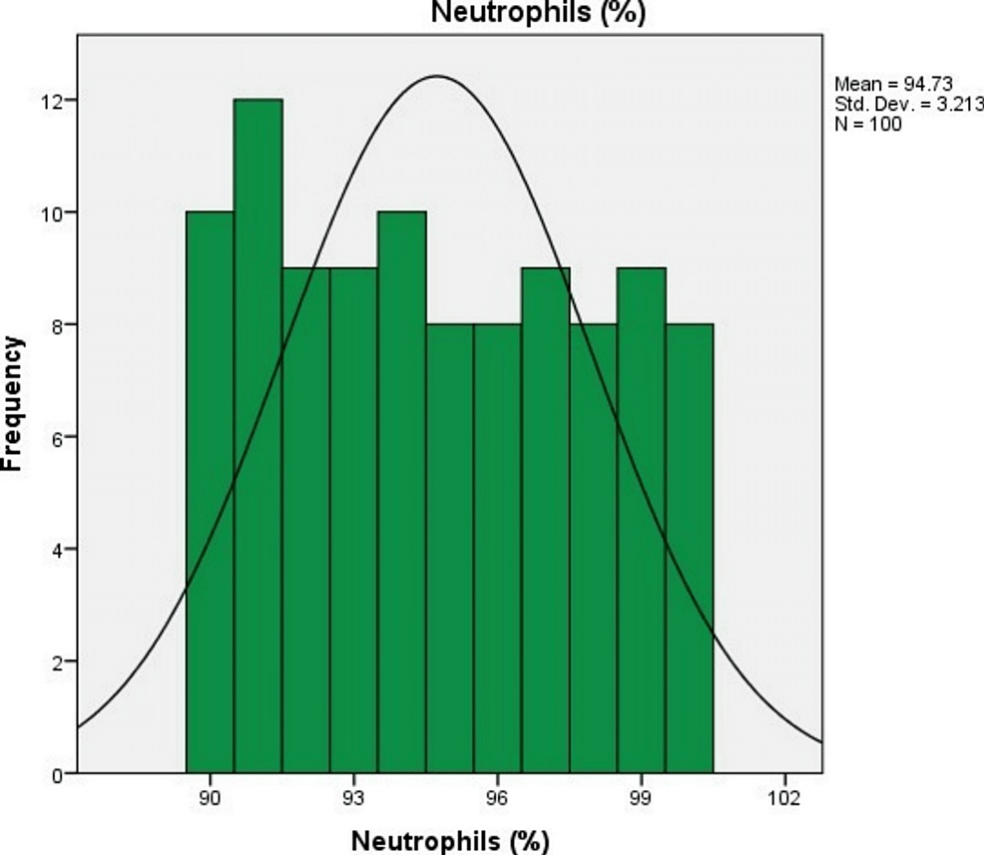
Neutrophil count.

**Figure 2:**
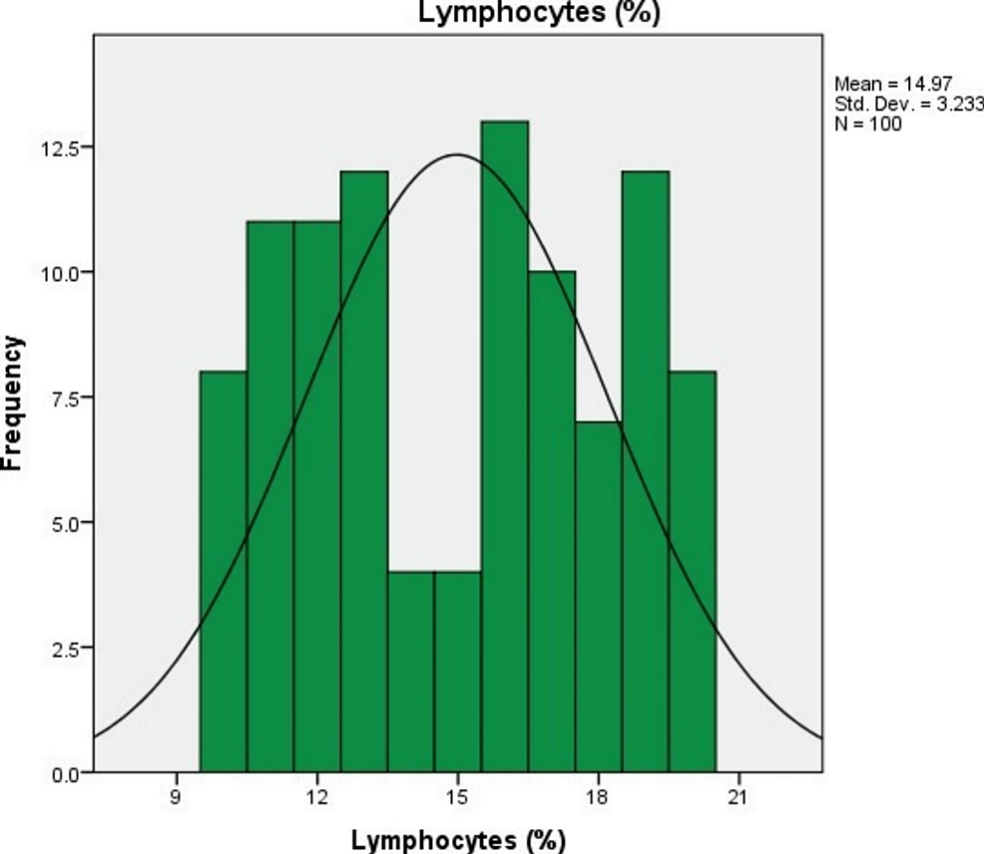
Lymphocyte count.

**Figure 3:**
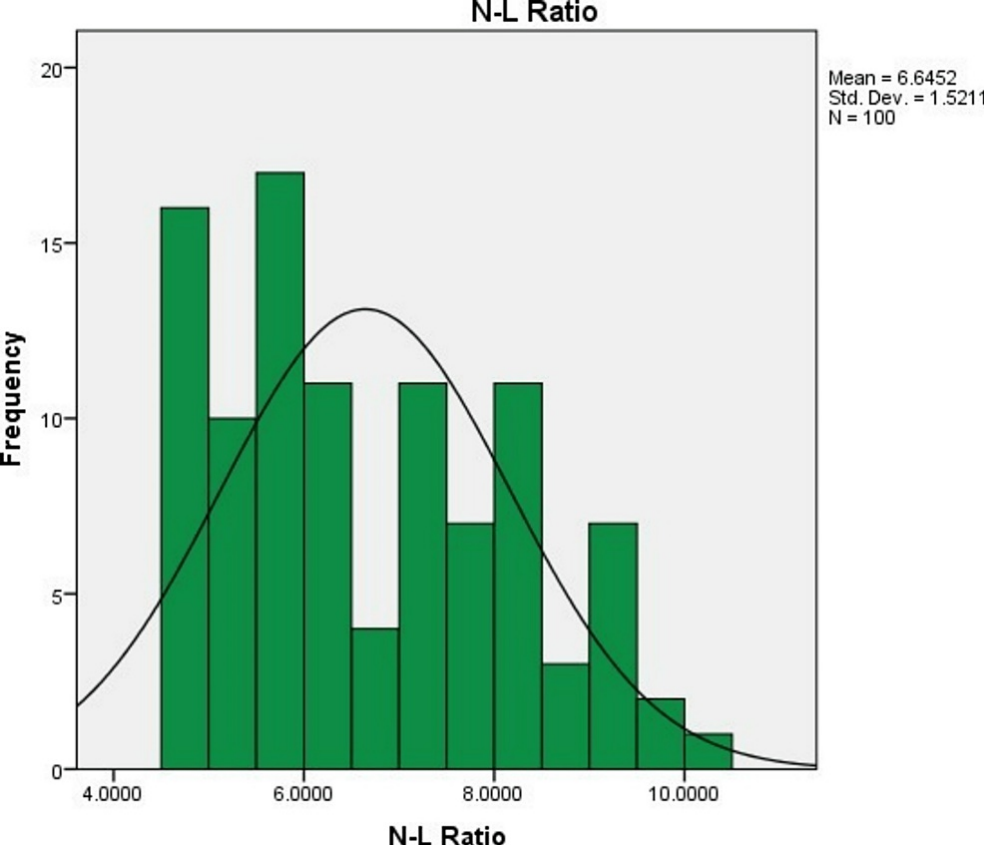
Neutrophil to lymphocyte ratio.

In 100 study subjects, the mean (SD) levels of neutrophils, lymphocytes, and the neutrophil-lymphocyte ratio were 94.73% (3.21%), 14.97% (3.23%), and 6.65% (1.52%), respectively. The minimum neutrophil percentage was 90%, and the maximum was 100%. The minimum lymphocyte percentage was 10%, and the maximum was 20%. The minimum NLR was 4.5, and the maximum was 10 (Figures 1-3 and Table 2).

**Table 2:** Descriptive statistics of neutrophils, lymphocytes, and neutrophil-lymphocyte ratio.

Descriptive statistics	Neutrophils (%)	Lymphocytes (%)	Neutrophil-lymphocyte ratio
Mean	94.73	14.97	6.645
Median	94.50	15.50	6.188
Mode	91	16	4.5
Standard deviation	3.21	3.23	1.521
Minimum	90	10	4.5
Maximum	100	20	10.00

**Figure 4:**
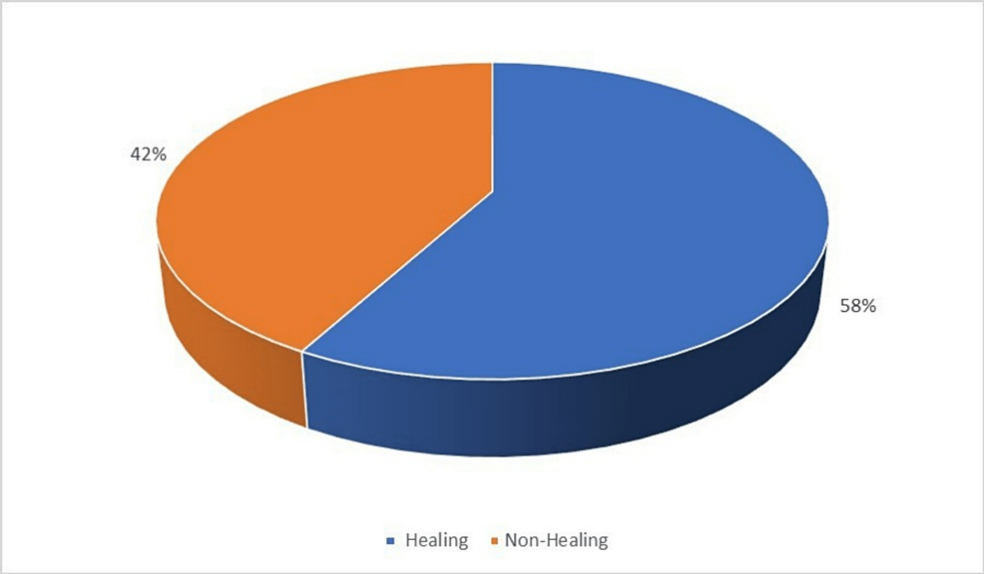
Outcome of diabetic foot ulcer among study subjects.

Majority of the diabetic foot ulcers were in healing stage after six weeks which can be indicated as a good prognostic sign (Figure 4).

**Figure 5:**
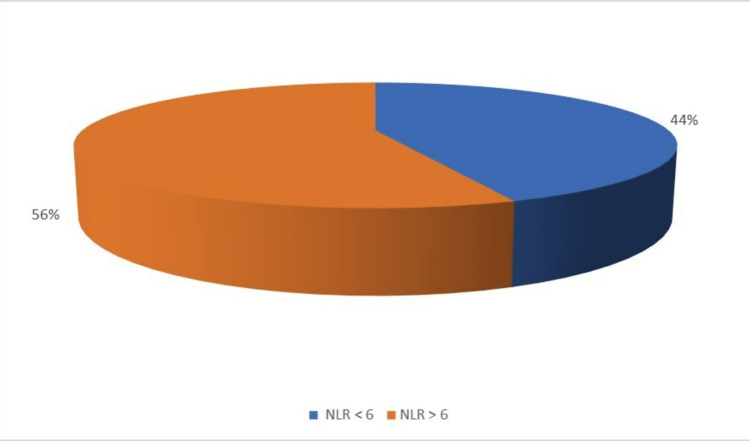
Distribution of neutrophil-lymphocyte ratio.

Among 100 patients, 56% of them have NLR >6, and 44% have NLR <6 (Figure 5).

There was a greater increase in NLR in non-healing ulcers than in healing ulcers, which was statistically significant (Table 3).

**Table 3:** The mean neutrophil-lymphocyte ratio between healing and non-healing ulcer.

Wound-healing/non-healing	N	Neutrophil-lymphocyte ratio mean ± SD	t-value	p-value
Healing	58	5.515 ± 0.647	3.41	<0.001
Non-healing	42	8.205 ± 0.84		

Forty-four (100%) of the study subjects with NLR <6 had the outcome of healing ulcers, while 42 (75%) of the subjects with NLR >6 progressed to non-healing ulcers, which was statistically significant (p-value = 0.001) (Table 4).

**Table 4:** Comparison of neutrophil-lymphocyte ratio and the outcome of diabetic foot ulcers among study subjects.

NLR	Non-healing ulcer	Healing ulcer	Chi-square value	p-value	Screening characteristics	
NLR > 6	42	14	56.89	0.001	Sensitivity: 100%	Positive predictive value: 75%
NLR < 6	0	44			Specificity: 75.9%	Negative predictive value: 100%

## Discussion

In the present study, the outcome of diabetic foot ulcers was 58% healing ulcers and 42% non-healing ulcers. NLR was ≥6 in 56% of the study population and <6 in 44% of the study population. 100% of non-healing ulcers and 25% of healing ulcers have an NLR of ≥6, and the mean NLR was higher in non-healing ulcers than in healing ulcers, which was significant. A study conducted by Kahraman et al. [4] had shown that there was a significant association between diabetic foot ulcers and NLR, representing a systemic inflammatory response. Elevated NLR of ≥5.25 was found to be an independently associated factor with shorter survival of the individual in a study [13]. Elevated NLR predicts worse amputation-free survival in patients with chronic critical limb ischemia [14]. A postoperative increase in NLR levels was found to be a reliable predictive biomarker of mortality in diabetic foot ulcer patients following amputation [15]. According to Vatankhah et al. [3], a larger NLR was linked to a higher likelihood of non-healing ulcers. Arican et al. [16] have observed that an NLR of <4.3 showed complete healing. Pierre-Louis et al. [17] showed that mortality within 30 days was higher in patients with high NLR in both the pre-operative and post-operative periods. Other similar studies also showed that there was a significant increase in the risk of amputation with high NLR [18,19].

In the present study, the sensitivity was 100%, the specificity was 75.9%, the positive predictive value was 75%, and the negative predictive value was 100%. According to Taşoğlu et al. [20], NLR 5.2 exhibited 83% sensitivity and 63% specificity in predicting amputation within 30 days of surgery and 63% sensitivity and 63% specificity in predicting midterm amputation.

Demirdal and Sen [21] showed that an NLR of >6.5 was calculated as the cut-off with 53.3% sensitivity and 63% specificity in predicting peripheral arterial disease and has a role in predicting amputations.

Limitations

Factors like platelet to lymphocyte ratio, wound size and wound ischemia, and different treatment modalities which determine the healing status of diabetic foot ulcers were not considered in the study.

## Conclusions

Our study shows that the NLR value depicts the outcome of diabetic foot ulcers. Study subjects with non-healing ulcers were having NLR > 6. There was a significant association between increased NLR and non-healing. NLR has high sensitivity as well as specificity and is a reliable indicator for determining the healing status of the ulcer. Hence, it can be used as a screening tool for determining the outcome of a diabetic foot ulcer. Further research should be done, including on other grades of diabetic foot ulcers and for a longer duration to assess the final outcome of a diabetic foot ulcer: complete healing, minor amputation, or major amputation.

## References

[1] Sapra A, Bhandari P (2022). Diabetes Mellitus. https://www.ncbi.nlm.nih.gov/books/NBK551501/.

[2] Gary T, Pichler M, Belaj K (2013). Neutrophil-to-lymphocyte ratio and its association with critical limb ischemia in PAOD patients. PLoS One.

[3] Vatankhah N, Jahangiri Y, Landry GJ, McLafferty RB, Alkayed NJ, Moneta GL, Azarbal AF (2017). Predictive value of neutrophil-to-lymphocyte ratio in diabetic wound healing. J Vasc Surg.

[4] Kahraman C, Yumun G, Kahraman NK, Namdar ND, Cosgun S (2014). Neutrophil-to-lymphocyte ratio in diabetes mellitus patients with and without diabetic foot ulcer. Eur J Med Sci.

[5] Alexiadou K, Doupis J (2012). Management of diabetic foot ulcers. Diabetes Ther.

[6] Howard R, Kanetsky PA, Egan KM (2019). Exploring the prognostic value of the neutrophil-to-lymphocyte ratio in cancer. Sci Rep.

[7] Kim S, Eliot M, Koestler DC, Wu WC, Kelsey KT (2018). Association of neutrophil-to-lymphocyte ratio with mortality and cardiovascular disease in the Jackson Heart Study and modification by the Duffy antigen variant. JAMA Cardiol.

[8] Angkananard T, Anothaisintawee T, McEvoy M, Attia J, Thakkinstian A (2018). Neutrophil lymphocyte ratio and cardiovascular disease risk: a systematic review and meta-analysis. Biomed Res Int.

[9] Tan YG, Sia J, Huang HH, Lau WK (2019). Neutrophil-to-lymphocyte ratio independently predicts advanced pathological staging and poorer survival outcomes in testicular cancer. Investig Clin Urol.

[10] Lou M, Luo P, Tang R, Peng Y, Yu S, Huang W, He L (2015). Relationship between neutrophil-lymphocyte ratio and insulin resistance in newly diagnosed type 2 diabetes mellitus patients. BMC Endocr Disord.

[11] Shiny A, Bibin YS, Shanthirani CS (2014). Association of neutrophil-lymphocyte ratio with glucose intolerance: an indicator of systemic inflammation in patients with type 2 diabetes. Diabetes Technol Ther.

[12] Shah P, Inturi R, Anne D (2022). Wagner’s classification as a tool for treating diabetic foot ulcers: our observations at a suburban teaching hospital. Cureus.

[13] Spark JI, Sarveswaran J, Blest N, Charalabidis P, Asthana S (2010). An elevated neutrophil-lymphocyte ratio independently predicts mortality in chronic critical limb ischemia. J Vasc Surg.

[14] González-Fajardo JA, Brizuela-Sanz JA, Aguirre-Gervás B, Merino-Díaz B, Del Río-Solá L, Martín-Pedrosa M, Vaquero-Puerta C (2014). Prognostic significance of an elevated neutrophil-lymphocyte ratio in the amputation-free survival of patients with chronic critical limb ischemia. Ann Vasc Surg.

[15] Chen W, Chen K, Xu Z (2021). Neutrophil-to-lymphocyte ratio and platelet-to-lymphocyte ratio predict mortality in patients with diabetic foot ulcers undergoing amputations. Diabetes Metab Syndr Obes.

[16] Arıcan G, Kahraman HÇ, Özmeriç A, İltar S, Alemdaroğlu KB (2020). Monitoring the prognosis of diabetic foot ulcers: predictive value of neutrophil-to-lymphocyte ratio and red blood cell distribution width. Int J Low Extrem Wounds.

[17] Pierre-Louis WS, Bath J, Mikkilineni S, Scott MC, Harlander-Locke M, Rasor Z, Smeds M (2019). Neutrophil to lymphocyte ratio as a predictor of outcomes after amputation. Ann Vasc Surg.

[18] Altay FA, Kuzi S, Altay M (2019). Predicting diabetic foot ulcer infection using the neutrophil-to-lymphocyte ratio: a prospective study. J Wound Care.

[19] Serban D, Papanas N, Dascalu AM (2021). Significance of neutrophil to lymphocyte ratio (NLR) and platelet lymphocyte ratio (PLR) in diabetic foot ulcer and potential new therapeutic targets. Int J Low Extrem Wounds.

[20] Taşoğlu I, Çiçek OF, Lafci G (2014). Usefulness of neutrophil/lymphocyte ratio as a predictor of amputation after embolectomy for acute limb ischemia. Ann Vasc Surg.

[21] Demirdal T, Sen P (2018). The significance of neutrophil-lymphocyte ratio, platelet-lymphocyte ratio and lymphocyte-monocyte ratio in predicting peripheral arterial disease, peripheral neuropathy, osteomyelitis and amputation in diabetic foot infection. Diabetes Res Clin Pract.

